# Supplementary material for: Quantitative 3D histochemistry reveals region-specific amyloid-β reduction by the antidiabetic drug netoglitazone
Source: PLoS One. 2025 May 6;20(5):e0309489. doi: 10.1371/journal.pone.0309489 (PMC12054868; doi:10.1371/journal.pone.0309489)
Supplement: S1 Fig — (DOCX) [file pone.0309489.s001.docx]

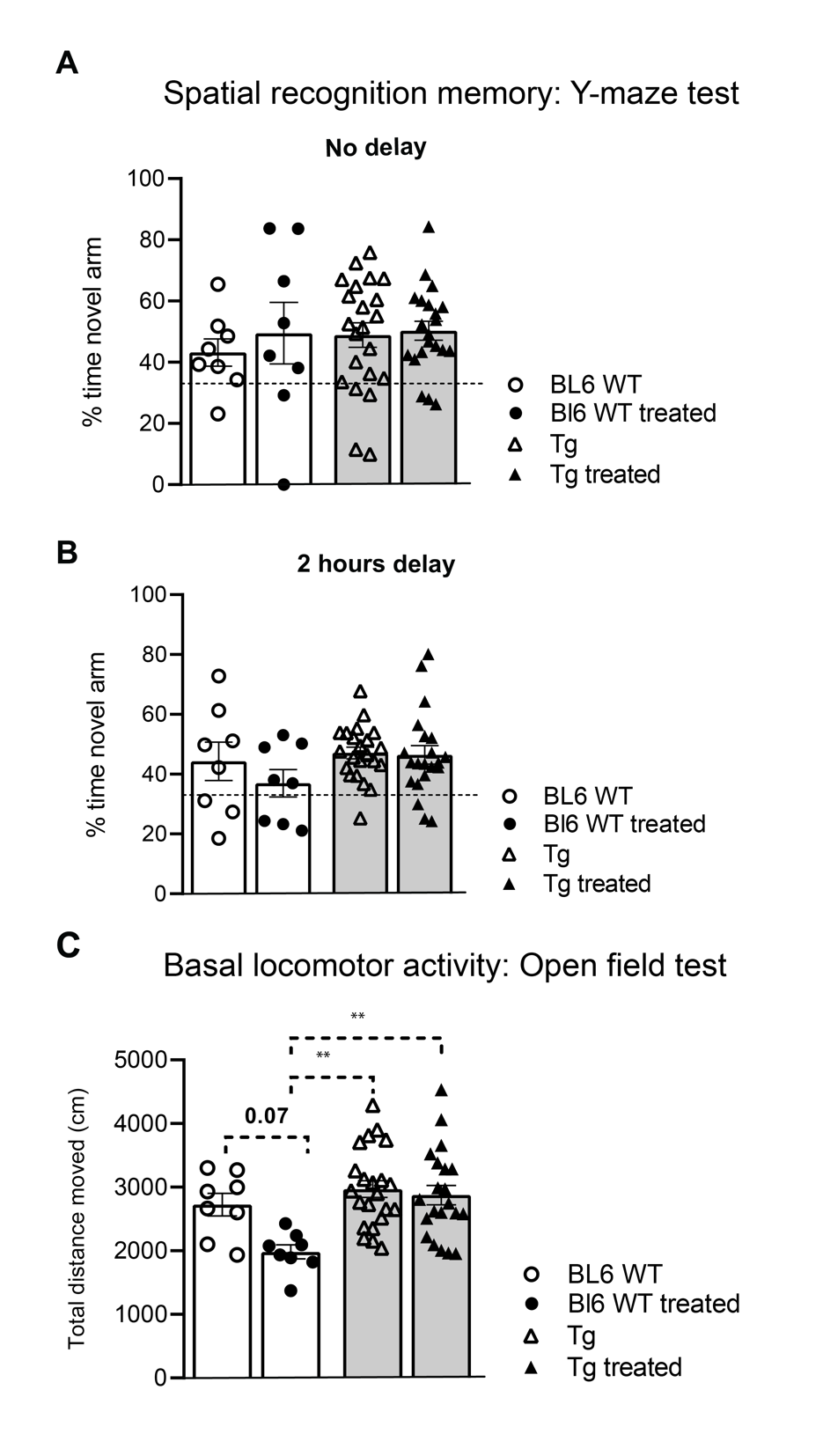


**S1 Fig:** **180 days of treatment with netoglitazone does not improve spatial recognition memory and basal locomotor activity. (A,B)** Behavioral tests. Spatial recognition memory: No difference was detected in time spent in the novel arm between treated and non-treated mice, independent on the genotype. The result was identical for both test delay times: no delay (A) or 2 hours delay (B). **(C)** Basal locomotor activity: Drug treatment did not affect basal locomotor activity in Tg mice as measure by the total distance moved in the arena. A difference was only observed when comparing Tg treated and Tg non-treated mice with WT treated mice.
