# Supplementary material for: Quantitative 3D histochemistry reveals region-specific amyloid-β reduction by the antidiabetic drug netoglitazone
Source: PLoS One. 2025 May 6;20(5):e0309489. doi: 10.1371/journal.pone.0309489 (PMC12054868; doi:10.1371/journal.pone.0309489)
Supplement: S2 Fig — (DOCX) [file pone.0309489.s002.docx]

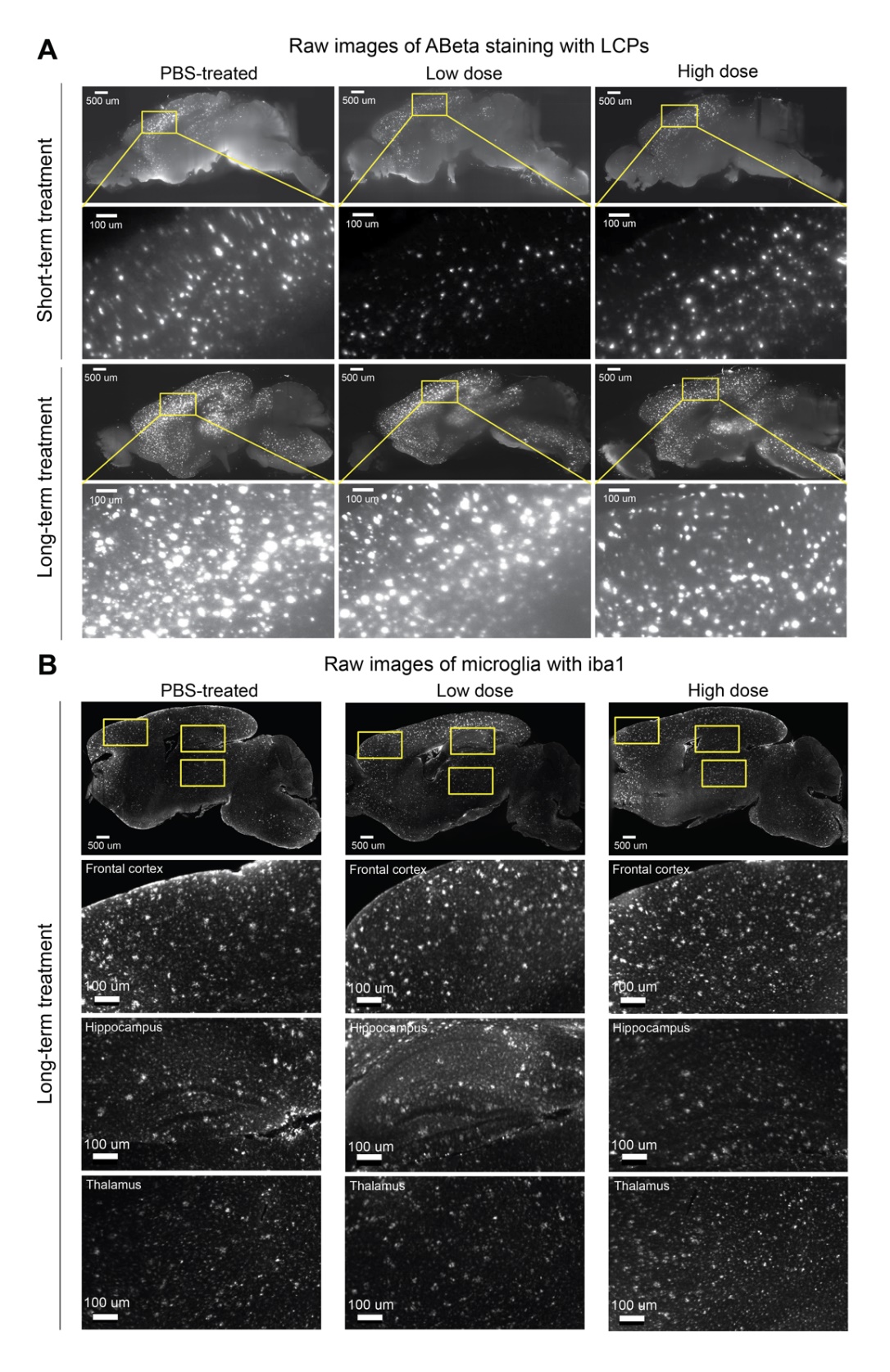


**S2 Fig.: Raw data of Aβ plaques and microglia staining.**  **(A)** Abeta staining with LCPs for plaque visualization in the whole-brain. Brains were cleared with focused electrophoretic tissue clearing (FEC). Representative raw images of sagittal brains slices of short and chronic treatment cohorts, and treatment dosages with respective controls. Zoom shows a part of the cerebral cortex where large amounts of amyloid plaques are normally present. **(B)** Microglia staining with anti-Iba1 antibody in the whole brain. Brains were cleared with a modified version of iDISCO. Representative raw images of sagittal brain slices of the high- and low-dose chronic treatment cohort and respective control. Zooms show frontal cortical, hippocampal, and thalamic regions where different volumes of microglia are detected.
