# Supplementary material for: Quantitative 3D histochemistry reveals region-specific amyloid-β reduction by the antidiabetic drug netoglitazone
Source: PLoS One. 2025 May 6;20(5):e0309489. doi: 10.1371/journal.pone.0309489 (PMC12054868; doi:10.1371/journal.pone.0309489)
Supplement: S3 Fig — (DOCX) [file pone.0309489.s003.docx]

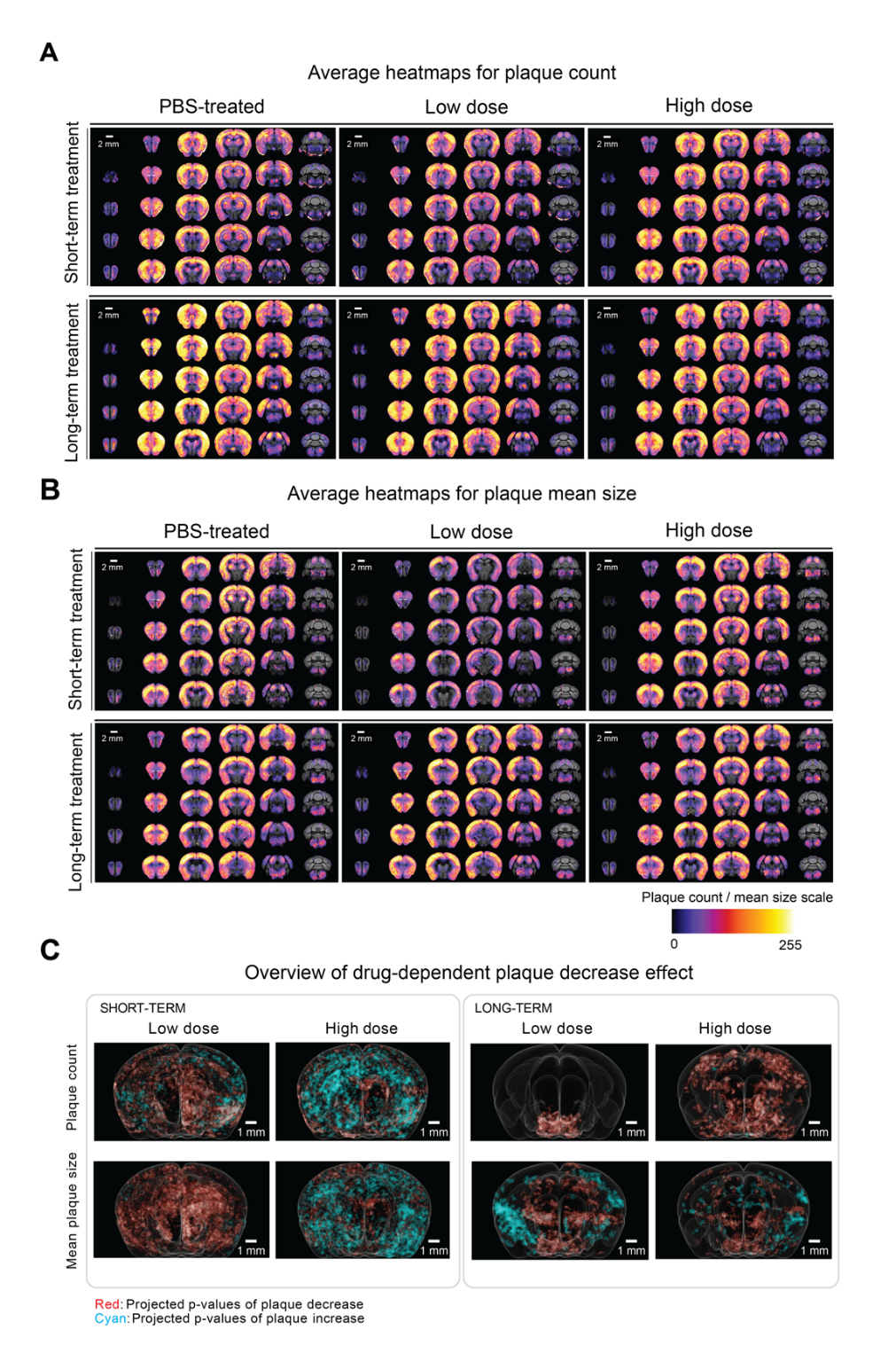


**S3 Fig.: Average number and size of plaque in the different cohorts. (A,B)** The mean plaque count and mean size for both, short and chronic treatments, were counted at each voxel in the atlas space and smoothed heatmaps were generated for control and treated brains. Figures show coronal sections of the whole brain where the difference count and mean size between groups can be visually appreciated and anticipates the anatomically patchy efficacy of the drug. In yellow and dark purple, the highest and lowest average of counts and mean sizes are depicted in each coronal section respectively. **(C)** A summary for the average effect of Netoglitazone in plaque decrease/increase per cohort. Figures summarize the 3-dimensional maps of statistically affected voxels (Figures 2 and 3 of the manuscript), by applying a projection over the depth of the brains across the coronal axis. A projection of the reference atlas is depicted in grey. This representation highlights with bright red/cyan colors the voxels where there is a significant effect (decrease/increase respectively) over the entire depth of the sample.
