# Supplementary material for: Quantitative 3D histochemistry reveals region-specific amyloid-β reduction by the antidiabetic drug netoglitazone
Source: PLoS One. 2025 May 6;20(5):e0309489. doi: 10.1371/journal.pone.0309489 (PMC12054868; doi:10.1371/journal.pone.0309489)
Supplement: S4 Fig — (DOCX) [file pone.0309489.s004.docx]

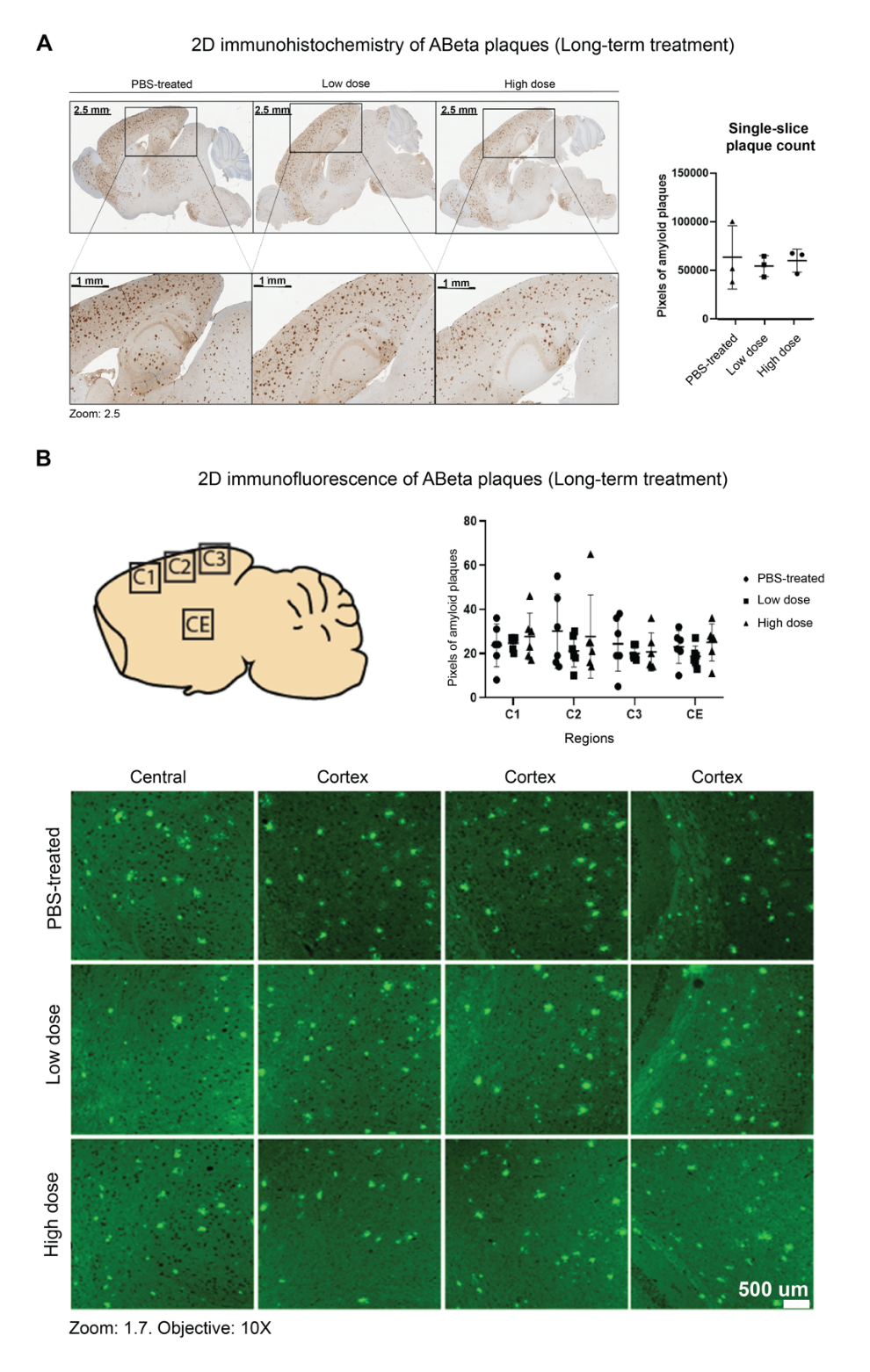


**S4 Fig.: 2D immunohistochemistry and immunofluorescence of Aβ plaques.** **(A)** Immunohistochemistry of Abeta plaques with anti-Abeta antibody in 10uM paraffin sections of the old cohort. Representative raw images of sagittal brain slices for each treatment dosage and respective controls: zoom 2.5X shows a part of the cerebral cortex and hippocampus. No statistical difference in plaque count was detected among treatments when counting for total pixels covered by plaques in the whole sections. **(B)** Immunofluorescence of Abeta plaques with anti-Abeta antibody in four different brain areas from 10uM paraffin sections of the old cohort. Representative raw images of three areas of the cerebral cortex (one at the border with the hippocampus) and one thalamic area for each treatment dosage and respective controls. 1.7X zoom and 10X objective were used. No statistical difference in plaque count was detected among treatments when counting for total pixels covered by plaques in the different areas.
