# Supplementary material for: Quantitative 3D histochemistry reveals region-specific amyloid-β reduction by the antidiabetic drug netoglitazone
Source: PLoS One. 2025 May 6;20(5):e0309489. doi: 10.1371/journal.pone.0309489 (PMC12054868; doi:10.1371/journal.pone.0309489)
Supplement: S5 Fig — Representative images depicting the quality of microglia segmentation. (DOCX) [file pone.0309489.s005.docx]

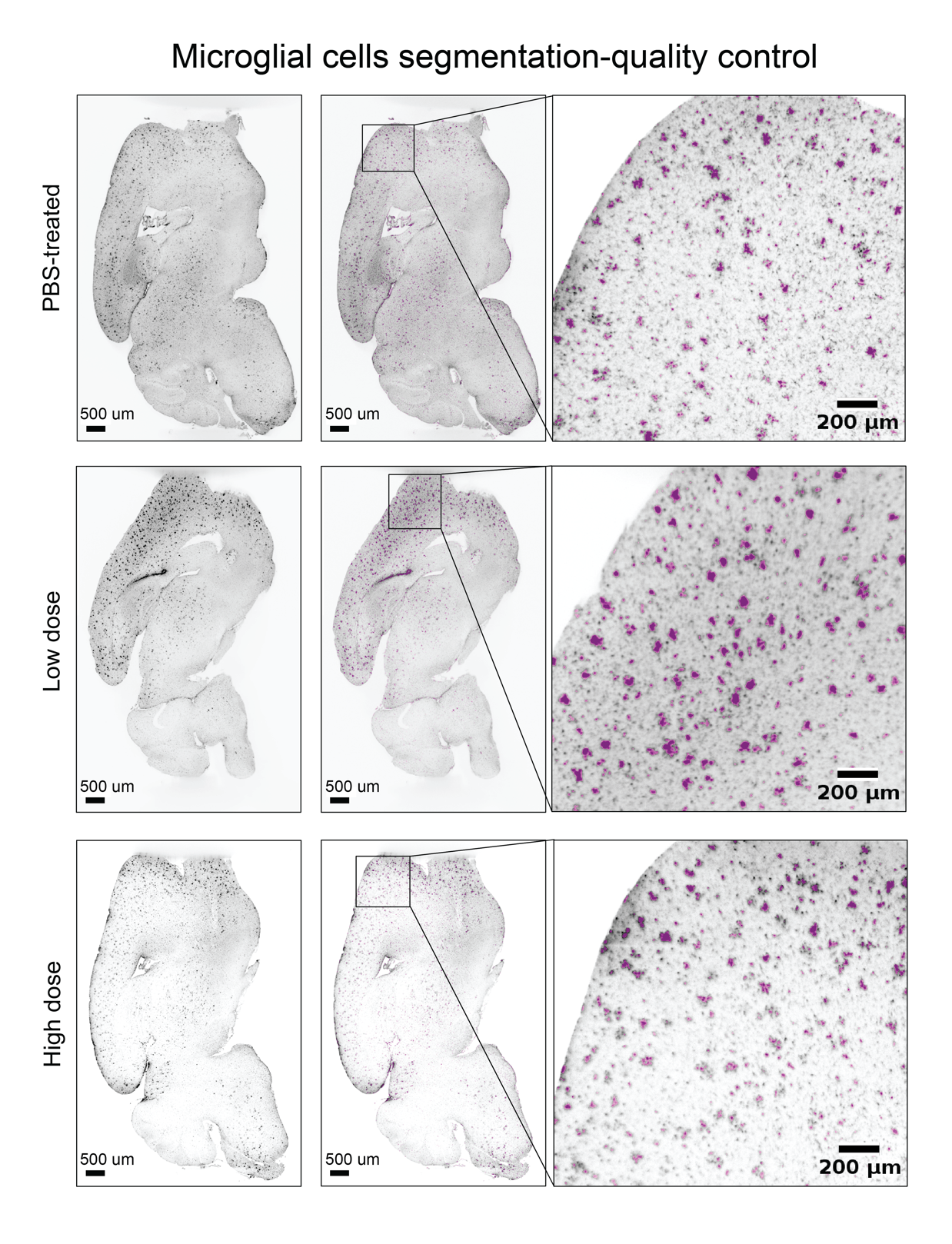


**S5 Fig.: Segmentation of microglia cells. Representative images depicting the quality of microglia segmentation.** A slice from the raw data is shown in the left column. An overlay of the segmented microglia in magenta, visualized over the raw data (grayscale) is shown in the middle column. A zoom-in on the cortex region (right column) is shown to provide detail.
