# Supplementary material for: Quantitative 3D histochemistry reveals region-specific amyloid-β reduction by the antidiabetic drug netoglitazone
Source: PLoS One. 2025 May 6;20(5):e0309489. doi: 10.1371/journal.pone.0309489 (PMC12054868; doi:10.1371/journal.pone.0309489)
Supplement: Attachment — Submitted filename: Response to Reviewers.pdf [file pone.0309489.s020.pdf]

PLOS ONE Reviewer comments from 10.03.2025

Reviewer #1: The authors have addressed the comments sufficiently. Perhaps two minor additions that could improve the discussion as the RNA sequencing data was not discussed/elaborated on as much:

1. Microglia are involved in plaque seeding via APOE (PMID: 39639016, 39419029), hence the changes in amyloidosis by netoglitazone could be mediated by microglial-mediated plaque formation. This could additionally explain the results of the high-dose netoglitazone observations as less microglia and plaques were observed. Additionally, since high-dose netoglitazone was shown to elevate CD68, the authors could argue using this data in the discussion to support their claim of elevated Aβ clearance.

2. The elevated Serpina3n is also interesting as it is a marker for disease-associated astrocytes (PMID: 32341542) and oligodendrocytes (PMID: 35760863) which could be relevant in other disease models where Aβ is not just produced by neurons or also in human cases as oligodendrocytes have recently been reported to contribute to Aβ production (PMID: 39103558).

Answer:
We sincerely appreciate the reviewer's insightful comments. In response, we have incorporated the suggested additions beginning at line 883 (in the Manuscript with track changes) and 821 (in the Manuscript) of the discussion, expanding on microglial involvement in plaque formation, the role of CD68 in Aβ clearance, and the relevance of Serpina3n to astrocytes, oligodendrocytes, and Aβ production. These valuable suggestions have further strengthened our manuscript, and we are grateful for the reviewer's constructive feedback.

Response to Journal Requirements
We have thoroughly reviewed and updated the reference list to ensure accuracy, completeness, and clarity. The following revisions have been made:
Added:

- References 106-112 to support the recently incorporated section in the discussion.
- Nishiyama, K., et al., *Presenilin 1 mRNA expression in hippocampi of sporadic Alzheimer's disease patients. Neurosci Res*, 1996. *26(1): p. 75-8*, to enhance clarity.
- Zhang, Y., et al., *Amyloid β-based therapy for Alzheimer's disease: challenges, successes, and future. Signal Transduction and Targeted Therapy, 2023. 8(1): p. 248*, to strengthen the discussion.

Removed:

- *The Human Protein Atlas*, which was mistakenly included and was not necessary in the context. We apologize for the oversight.

- Schalbetter, S.M., et al., *Adolescence is a sensitive period for prefrontal microglia to act on cognitive development. Science Advances, 2022*, as it was considered non-essential.

Replaced:

- Rafii, M.S., & Aisen, P.S., *Brain region-specific pharmacodynamics. Alzheimer's & Dementia, 2009* → Replaced with **Srinivas, N., K. Maffuid, and A.D.M. Kashuba, *Clinical Pharmacokinetics and Pharmacodynamics of Drugs in the Central Nervous System. Clin Pharmacokinet, 2018. 57(9): p. 1059-1074*** for a more recent and relevant reference.
- HABCHI, J.Y., et al., *THERAPY FOR PROTEIN MISFOLDING DISEASE, W.T. LIMITED, 2019* → Replaced with **Habchi, J., et al., *Systematic development of small molecules to inhibit specific microscopic steps of Aβ42 aggregation in Alzheimer's disease. PNAS, 2017. 114(2): p. E200-E208*** for improved citation accuracy.

Resolved: All reference repetitions have been resolved.

We appreciate PlosOne guidance in refining our reference list and remain open to any further adjustments as needed.
