# Supplementary material for: Quantitative 3D histochemistry reveals region-specific amyloid-β reduction by the antidiabetic drug netoglitazone
Source: PLoS One. 2025 May 6;20(5):e0309489. doi: 10.1371/journal.pone.0309489 (PMC12054868; doi:10.1371/journal.pone.0309489)
Supplement: Attachment — Submitted filename: Response to Initial Review.docx [file pone.0309489.s018.docx]

**Response to Initial Review** **Manuscript ID: PONE-D-24-31056** **Title: Quantitative 3D histochemistry reveals region-specific amyloid-β reduction by the antidiabetic drug netoglitazone**

Dear Dr. Miriam Ann Hickey,

We would like to thank you for your thoughtful evaluation of our manuscript. We appreciate the opportunity to revise and improve our work. Below, we provide detailed responses to each of the points raised by the academic editor and reviewers. We have made the necessary revisions to the manuscript accordingly.

**Addressed Comments:**

1. **Supplementary figures (the paper makes reference to at least 4) appear to be missing.**

We sincerely apologize for the oversight in the initial submission. It appears that the figures file was mistakenly uploaded instead of the supplementary figures file. We have now corrected this mistake and uploaded the appropriate supplementary figures in the revised submission (S1-S5 Figs.).

2. **Reference to figure 5 (lines 731 - 733) appears incorrect.**

Thank you for pointing out this error. We have reviewed the reference and corrected it in the revised manuscript. The reference now correctly points to Figure 4, aligning with the relevant content in the text. The references to the figures are now located on lines 481 and 483.

3. **The number of mice within each group is insufficiently clear, as there appears to be differences in group size depending upon the behavioural outcome measure, e.g., Figure 1C versus Figure 1D. The number of males and females within each group is insufficiently clear.**

We appreciate the reviewer’s observation regarding the clarity of the number of mice used in each group. To address this concern, we have created a detailed table (Table 1) that provides a clear breakdown of the number of mice used for each figure, including the specific group sizes and the distribution of males and females. The table has been added after the paragraph that cites it on line 123 in the 'Materials and Methods – APPPS1 Mice' chapter of the revised manuscript to ensure full transparency and clarity.

Due to an established sex difference in freezing responses as the indicator of conditioned fear (Figure 1D), with alternative behavioral responses being present primarily in females (Gruene TM, Flick K, Stefano A, Shea SD, Shansky RM. 2015. Sexually divergent expression of active and passive conditioned fear responses in rats. Elife 4: e11352. 10.7554/eLife.1135), we herein focused on male animals in the assessment of fear memory, resulting in the use of fewer animals overall. We have added a few lines of clarification starting from line 269.

4. **There appears to be regional sidedness in the pattern to reduced plaques, and discussion of this is insufficient.**
   - **e.g., the ventrolateral cerebellum left side only (possibly paraflocculus, Fig 2A, images on far right).**
   - **e.g., right piriform cortex and right frontal cortex and olfactory tubercle (Figure 2A, images on left, 2nd and 3rd columns).**
   - **e.g., right caudal hippocampus (Figure 2A, middle column, middle right column).**

We appreciate the reviewer’s insightful comments on the regional asymmetry in plaque reduction. In response, we have expanded our discussion in the revised manuscript to explore these regional differences in greater detail. We now include a dedicated section, starting from line 788 that examines potential reasons for this asymmetry, such as varying drug distribution, regional differences in amyloid pathology, and the natural asymmetry in brain structure and function. These factors may impact the region-specific effectiveness of netoglitazone.

5. **Differences between 3D histochemistry and traditional 2D immunohistochemical results are insufficiently discussed in the body of the manuscript.**

We appreciate the reviewer’s comment highlighting the need for a more detailed discussion on the differences between 3D histochemistry and traditional 2D immunohistochemistry. In response, we have expanded the discussion section of the manuscript to address this comparison more thoroughly.

Specifically, we have added a paragraph that directly contrasts the capabilities of these two methodologies. While traditional 2D immunohistochemistry offers valuable insights, it is inherently limited by its slice-based perspective, which can result in sampling biases and an incomplete understanding of plaque distribution across the brain. In contrast, 3D histochemistry allows for whole-brain imaging, providing a more comprehensive view of plaque burden and enabling the detection of regional differences and asymmetries that might be overlooked in 2D analyses. This addition is located in the discussion, immediately after the paragraph where we discuss the advantages of Q3D in detecting both favorable and unfavorable changes in amyloid quantity, starting from line 836, thereby enhancing the overall discussion on the methodologies used in our study. Additionally, we have included a supplementary figure (S4 Fig) in the revised submission, which was previously omitted due to an upload error. This figure may provide further clarity in understanding these distinctions. We apologize for the oversight.

6. **Within the body of the re-submitted manuscript, there are numerous examples of this text: "Error! Reference source not found".**

Thank you for bringing this issue to our attention. We sincerely apologize for the oversight. We have carefully reviewed the manuscript and corrected all instances of "Error! Reference source not found" by properly adjusting the references throughout the document.

**Conclusion:**

We have addressed all of the points raised during the review process and believe that these revisions have strengthened our manuscript. We hope that the revised manuscript meets the publication criteria of PLOS ONE and look forward to your feedback.

Thank you for your consideration.

Sincerely,

**Dr. Francesca Catto**

Wagistrasse 18, 8952, Schlieren

Zurich, Switzerland

[catto@imai.c](mailto:catto@imai.ch)h

[www.imai-medtech.com/](https://www.imai-medtech.com/)
