# Supplementary material for: Quantitative 3D histochemistry reveals region-specific amyloid-β reduction by the antidiabetic drug netoglitazone
Source: PLoS One. 2025 May 6;20(5):e0309489. doi: 10.1371/journal.pone.0309489 (PMC12054868; doi:10.1371/journal.pone.0309489)
Supplement: Attachment — Submitted filename: Response to Reviewers.docx [file pone.0309489.s019.docx]

PLOS ONE Reviewer comments. 04.12.2024

**Reviewer #1:** **The authors performed experiments to assess the modulatory effects of netoglitazone on Aβ plaque burden in vivo. The authors proposed that netoglitazone administration reduces Aβ plaque deposition and highlights the strength of volumetric assessment of Aβ plaque pathology in preclinical AD research. While the addition of information regarding asymmetrical plaque deposition is intriguing, there are still some concerns regarding the data presentation of the manuscript in its current form. See comments below:**

**1. Introduction**

**Regarding the amyloid cascade hypothesis, could the authors comment on the detrimental role that Aβ oligomers also play in inducing neuronal deficits? Although plaques seemingly exert physical detriments to the parenchyma and cells proximal to them, it remains debated whether an overabundance of oligomeric Aβ not bound in plaques would be more detrimental to neural functions. This could also explain why Aβ plaque deposition does not correlate well with cognitive decline, as it could be possible that plaques act as a reservoir for oligomeric Aβ from disrupting synaptic and other neural functions.**

We thank the reviewer for this insightful observation. To address this point, we have added a section starting at line 67 of both the “Revised Manuscript with Track Changes” and the “Manuscript” in the introduction, which discusses the role of soluble Aβ oligomers in AD pathogenesis. Specifically, we highlighted how oligomers are highly neurotoxic. Additionally, we introduced the multifaceted role of plaques, acknowledging their contributions to localized tissue damage and neuroinflammation, as well as their potential to act as reservoirs for Aβ oligomers. This interplay between plaques and oligomers underscores their complexity as both drivers and modulators of AD pathology. References [6, 7] have been included to support this addition.

**2. Methods:**

**a. Could the authors provide details on how the APPPS1 mice were bred? For instance, what is the sex of the transgene carrier for all breeding? A consistent reporting of breeding schemes would benefit readers and encourage transparency and reproducibility. Moreover, the usage of the term gender should be replaced with sex.**

We thank the reviewer for this important suggestion. We have now clarified the breeding scheme used for the APPPS1 mice. Specifically, 68% of the transgene-carrying parents were male, while 32% were female. We added a sentence in line 130 of both the “Revised Manuscript with Track Changes” the “Manuscript” to explicitly describe the breeding scheme for clarity and reproducibility. Additionally, we have revised the manuscript to replace the term “gender” with “sex” to align with standard biological terminology.

**b. Could the authors please indicate the different sex of animals (different symbols) used in graphs where pooling of male and female animals was performed? The authors noted in this revised manuscript that sex difference in freezing responses occur, but could the authors also comment on whether sex dimorphism in Aβ plaque load is evident between male and female APPPS1 mice? If sex dimorphism is evident, should the assessment of plaque load not be stratified further based on sex? In fact, the authors also openly stated in the discussion section that there is a disparity in PPARγ receptor expression between males and females.**

We sincerely appreciate the reviewer’s thoughtful comments regarding the representation of sex differences in our study. In response, we have now indicated the sex of the animals using different symbols in Figure 1. Triangles represent females, while circles represent males.

Regarding Aβ plaque load, while sex differences in freezing responses and PPARγ receptor expression are acknowledged in our manuscript, our primary objective is to assess the overall therapeutic effects of the intervention on Aβ plaque load and behavioural outcomes in the APPPS1 cohort as a whole. In the case of conditioned fear assessment, we focused on male animals due to well-established sex differences in freezing responses, which could introduce behavioural variability unrelated to the intervention itself. However, Aβ pathology in the APPPS1 model is influenced by multiple factors, and while stratifying plaque load by sex could provide additional insights, our study was not specifically designed to investigate sex dimorphism in Aβ pathology. Introducing such an analysis could add complexity that extends beyond the scope of our primary research question. Nonetheless, we recognize the importance of sex as a biological variable and appreciate the reviewer’s perspective, which may be valuable for future investigations.

**3. Results:**

**a. Figs 2 and 3 – The significant voxels and average values are very small and rather difficult to read. Is it possible to increase the size of these panels to improve legibility? Moreover, do early and late mean short-term (90 days) and long-term (180 days)? If so, the caption in the figures should be modified accordingly to improve clarity.**

We thank the reviewer for their valuable feedback regarding Figures 2 and 3.

**Panel Size:** We acknowledge the difficulty in reading the significant voxels and average values due to their small size. While we attempted to enlarge the panels in Figures 2 and 3 in the revised manuscript, further increasing the size proved challenging without disrupting the layout. We have made adjustments to improve clarity as much as possible and hope this version is suitable.

**Replacement of "Early" and "Late"**: In response to the reviewer’s comment, we have replaced the terms "early" and "late" with “low dose, 90 days” , “low dose, 180 days”, “high dose, 90 days”, and “high dose, 180 days” throughout the captions of Figures 2 and 3 for improved clarity.

**b. Figs 2 – Could the authors elaborate on how significant voxels sometimes do not correspond to similar statistical differences between treated and untreated groups in the average count? Should conclusions not be drawn from the average count instead? Lastly, does the average count not correspond with the 2D analysis in Fig S4 which shows unchanged plaque load?**

1. **Significant Voxels vs. Average Count**:

We appreciate the reviewer’s insightful question regarding the relationship between significant voxels and statistical differences observed in the average count and size of plaques. Voxel-based analysis provides a spatially resolved method for detecting regional differences in plaque distribution, allowing for localized statistical assessments that may not always align directly with aggregate measures. This discrepancy arises because voxel-wise comparisons account for spatial heterogeneity in plaque deposition, identifying localized effects that might be diluted when averaging across larger anatomical regions. In contrast, average plaque count comparisons across regions aggregate values over larger areas, potentially masking finer spatial patterns of significance that voxel-based analyses can detect. While the average count remains informative for overall cohort-level comparisons, voxel-based statistics provide additional granularity, highlighting spatially specific effects of treatment.

Thus, both measures provide complementary insights: average count offers a broader overview, while voxel-wise significance pinpoints localized treatment effects that might otherwise remain undetected.

**Correspondence with Fig. S4**:

We appreciate the reviewer’s question regarding the comparison between the 3D and 2D analyses. The difference in statistical outcomes between the two methods is primarily due to the resolution and comprehensiveness of the analyses.

In the 3D voxel-based analysis, significance is detected only in specific regions, while most other regions do not show statistical differences. This reflects a localized effect of the treatment rather than a global reduction in plaque load. In contrast, the 2D immunohistochemistry and immunofluorescence analysis provide a more limited sampling of brain regions, which may not capture these localized changes, leading to an overall non-significant result. Additionally, 3D analysis allows for a spatially resolved comparison across the entire brain volume, increasing sensitivity to detect regional effects that may not be apparent when averaging across larger anatomical structures in 2D. The statistical power of the 3D approach is therefore inherently different, as it considers voxel-level distributions rather than relying on sampled slices.

**c. Fig 3 – Could the authors comment on why the graphs on the right in this figure depict the average plaque count again as opposed to the average plaque size? Are the average plaque counts per region plotted to again show differences caused by the treatment in a separate cohort of animals? From the table listing the number of experimental animals, it seems that the same mice were used to generate the data from Fig 2 and Fig 3, but the average count values from both figures do not match. Could the authors please clarify?**

We thank the reviewer for pointing out these important issues and allowing us to clarify, and we sincerely apologize for any confusion caused.

Upon review, we realized that the legend for the graph in Fig. 3 labelled as "aggregate count" is incorrect and should instead read "mean aggregate size." We sincerely apologize for this oversight. The data depicted in the graphs on the right in Fig. 3 reflect the aggregate mean size per region, not the plaque count. This has been corrected in the revised figure legend to accurately represent the data. Regarding the cohort of animals, we confirm that the same mice were used to generate the data for both Fig. 2 and Fig. 3. The discrepancy in values between these figures is expected, as they represent different metrics: Fig. 2 shows plaque count, while Fig. 3 presents mean plaque size. We appreciate the reviewer’s careful attention to this detail and have revised the manuscript to ensure clarity and avoid any potential misunderstandings.

**d. Fig 4A – In terms of data presentation, the white outline of the brain subregions seems to be too thick and hence distracting from the colours annotating the significant increases and decreases. If possible, could the authors please reduce the outline thickness or reduce the number of regions annotated to only show major subregions as in Figs 2 and 3?**

We thank the reviewer for this valuable suggestion to improve the clarity of Fig. 4A. We have reduced the thickness of the white outlines in Fig. 4A to make the data presentation less distracting and ensure the colours annotating the significant increases and decreases are more prominent.

**e. Fig 5 – In the main text, the authors described an abundance of DEGs associated with the high-dose treatment while DEGs are limited in mice that received low-dose treatment. In the figure, however, this is the opposite. Could the authors please clarify?**

We appreciate the reviewer’s attention to detail and for pointing out this discrepancy. Upon review, we identified that the panels in Figure 5 were inadvertently switched. Panel A should correspond to the low-dose treatment, while Panel B represents the high-dose treatment. We sincerely apologize for the confusion caused by this labelling error in the figure legend. This correction ensures consistency between the figure and the main text, which states that the low-dose treatment resulted in a significant change, affecting 361 DEGs, whereas the high-dose treatment led to only a small change, with six DEGs being significantly altered.

**4. Discussion:**

**a. The authors stated that the hippocampus and the cortex are prone to amyloid deposition. However, this statement seems misleading as the high plaque load in these regions can be directly linked to regions of Thy1-promoter activity (PMID: 12112467), which is the promoter used in the APPPS1-Jucker line. Hence, high plaque load in these regions is presumably a direct effect of transgene expression in a subset of neurons in said regions.**

We thank the reviewer for this important observation. To address this, we have added a section starting from line 834 of the of the “Revised Manuscript with Track Changes” and line 773 of the “Manuscript” clarifying the factors contributing to regional differences in treatment efficacy and amyloid plaque distribution in the APPPS1-Jucker mouse model. This addition highlights the role of the Thy1-promoter in driving amyloid accumulation in the hippocampus and cortex, avoiding the potential misinterpretation that these regions are inherently prone to amyloid pathology. We believe this clarification improves the accuracy and transparency of the manuscript.

**Reviewer #2: This study aims to interrogate the effect of netoglitazone in an animal model of Alzheimer's disease. To evaluate its effect, the authors perform behavioural tests and whole-brain immunohistochemistry. The authors found changes in behaviour and a dose-dependent reduction on plaque density and microglial activation.**

**The technique used in this manuscript is novel and interesting. However, the manuscript is difficult to read due to the repetition of the methods section in the results. The data is properly discussed and the conclusion reached sustained by the results obtained. However, I have some concerns about this manuscript.**

**Major points:**

1- **The manuscript is too long. Although the three-dimensional histology must be explained in detail as it is the main point of this manuscript, the results section is impossible to understand. In all the subsections there is a long introduction repeating the information already stated in the methods section as well as, in some cases, a discussion of the results. This also occurs in the figure legends. This makes the manuscript tedious and distracts the interest of the reader. All this repeated information should be removed.**

We sincerely appreciate the reviewer’s feedback and understand the concern regarding the length and clarity of the results section. In response, we have carefully revised the manuscript to remove redundant information that was previously repeated from the methods section. We have streamlined the results section to focus on the key findings while ensuring that all necessary details for comprehension are retained.

**Minor points:**

**1- The authors state in the introduction that amyloid-PET has very low spatial resolution. Currently, the resolution of PET systems for rodents is under 1 mm, which in my opinion, is not low. Maybe the authors meant that the resolution is not enough to discriminate between plaques. This sentence should be rephrased.**

We thank the reviewer for this important clarification regarding the resolution of modern PET systems. We acknowledge that describing PET as having "very low spatial resolution" may be misleading, as current systems for rodents achieve sub-millimetre resolution, which is sufficient for regional analyses. However, our intended point was that this resolution remains inadequate for distinguishing individual amyloid plaques. To address this, we have rephrased the sentence starting from line 105 of both the “Revised Manuscript with Track Changes” and the “Manuscript” as follows: "While modern PET systems for rodents achieve sub-millimetre resolution, which is sufficient for regional analyses, their resolution remains insufficient to discriminate individual amyloid plaques." We believe this revision more accurately reflects the capabilities and limitations of PET systems and improves clarity. We appreciate the reviewer’s insightful comment, which has helped us refine our wording.

**2- The treatment is started before plaque deposition in this model. Thus, the effect observed is not a reduction on the plaques number but a prevention or deposition reduction.**

We thank the reviewer for raising this important point. In the first instance where the terminology for plaque decrease and plaque increase appears in the Results section, we have added a sentence starting from line 645 of the “Revised Manuscript with Track Changes” and line 611 of the “Manuscript” to clarify these terms: “Result of the reduction in the number of accumulated plaques, referred to as 'plaque decrease' from here on,” and similarly for increase: “Result of the augmentation in the number of accumulated plaques, referred to as 'plaque increase' from here on.” We hope this clarification helps ensure consistency in our terminology and addresses the reviewer’s concern.

**3- Behavioral results are almost not discussed. For example, netoglitazone increased freezing behavior also in WT animals. This is not further discussed. In addition, there is no correlation of these results to the histology measurements. Which was the objective of performing these tests then?**

We thank the reviewer for their insightful comment regarding the behavioural results and the need for further discussion. In response, we have expanded the discussion section starting from line 785 of the “Revised Manuscript with Track Changes” and line 724 of the “Manuscript” to better integrate the behavioural findings and their implications. We hope this addition clarifies the objectives of the behavioural tests and provides a more comprehensive discussion of their implications.

**4- The authors compare the 3D histology results to traditional 2D immunohistochemistry to show the superiority of the technique. However, there is no comparison to other quantitative technique, such as ELISA or even PET imaging, to validate the results.**

We thank the reviewer for this insightful question. The core focus of our study is the ability of our methods to detect microanatomical regional differences following netoglitazone treatment.

Whole-brain ELISA measurements, while valuable for quantifying total plaque burden, lack the necessary spatial resolution to capture localized effects. Even if ELISA were performed on micro-dissected neuroanatomical regions, this approach is technically not feasible due to the challenge of preserving regional integrity and ensuring precise sampling.

Similarly, PET imaging, despite its advantages in longitudinal in vivo tracking, is fundamentally limited in spatial resolution and, like ELISA, cannot resolve subtle, region-specific changes in plaque distribution. The spatial resolution constraints of PET imaging have been well-documented (Moses WW. Fundamental Limits of Spatial Resolution in PET. Nucl Instrum Methods Phys Res A. 2011 Aug 21;648 Supplement 1:S236-S240. doi: 10.1016/j.nima.2010.11.092. PMID: 21804677; PMCID: PMC3144741) making it unsuitable for detecting fine-scale anatomical effects. Therefore, while ELISA and PET serve as complementary methods for assessing overall plaque burden, they are not appropriate for validating our results, as they do not provide the necessary regional specificity. Our 3D histology approach remains the most suitable technique for detecting the localized treatment effects that are the focus of this study.
